# Systematic Co-Design of Artificial Intelligence-Enabled Innovations for Healthy Aging: A Demonstration Study Involving Socially Assistive Robots for Dementia Care

**DOI:** 10.3390/ijerph23081019

**Published:** 2026-08-04

**Authors:** Sajay Arthanat, Jing Wang, Dain LaRoche, Heather Fritz, Rosanne DiZazzo-Miller, Mostafa Hussein, Ola Ghattas, Moniruzzaman Akash, Momotaz Begum

**Affiliations:** 1 Department of Occupational Therapy, University of New Hampshire, Durham, NH 03824, USA; 2 School of Nursing, University of New Hampshire, Durham, NH 03824, USA; 3 Department of Kinesiology, College of Health and Human Services, University of New Hampshire, Durham, NH 03824, USA; 4 School of Occupational Therapy, Pacific Northwest University of Health Sciences, Yakima, WA 98901, USA; 5 Occupational Therapy Program, Wayne State University, Detroit, MI 48201, USA; 6 Department of Computer Science, University of New Hampshire, Durham, NH 03824, USA

**Keywords:** Alzheimer’s disease and related dementia, dementia, socially assistive robots, aging in place, co-design, Goal Attainment Scaling, Unified Theory of Acceptance and Use of Technology

## Abstract

**Highlights:**

**Public health relevance—How does this work relate to a public health issue?**

- Artificial intelligence-enabled technologies developed through systematic co-design can address growing public health challenge of dementia and caregiver burden
- This study demonstrates how artificial intelligence-enabled technologies such as socially assistive robots can potentially improve aging in place and health of older adults.

**Public health significance—Why is this work of significance to public health?**

- Demonstrates the importance of co-design that is inclusive and responsive to the needs of older adults with dementia and caregivers.
- Provides evidence that personalized smart home technology may enhance daily living for older adults with dementia and caregivers.

**Public health implications—What are the key implications or messages for practitioners, policy makers and/or researchers in public health?**

- Technology developers and researchers should consider actively engaging older adults and informal caregivers through co-design to improve usability and value of artificial intelligence-enabled technologies.
- Co-design enables iterative, scalable, and ethical development of artificial intelligence-enabled technologies for healthy aging in place.

**Abstract:**

Artificial intelligence-enabled technologies offer new opportunities to support healthy aging and the long-term care needs of older adults. However, inclusive practices are paramount to ensuring that accessibility, usability, privacy, and equitable use are factored into the design and deployment of these emerging technologies. This article explores the role of co-design in health technology development and highlights the application of three methodological tools—the NIH Stage Model for Behavioral Intervention Development, the Unified Theory of Acceptance and Use of Technology, and Goal Attainment Scaling—to create a smart-home-based socially assistive robot (SAR) for the care of individuals living with Alzheimer’s disease and related dementias (ADRD). Ten participants (five caregiver–care recipient dyads) from an ongoing mixed-methods pilot feasibility study trialed the SAR in their homes for 1–6 months, with the robot personalized to their daily functioning, home layout, and caregiving needs. Qualitative analysis of monthly interviews derived themes pertaining to technical design, care protocol design, training management, and complementary care. These themes, combined with goal attainment analysis, offered several insights that allowed us to iteratively scale and refine the technology tailored to ADRD care. The study offers a practical framework for future co-design efforts aimed at enhancing the adoption of AI-enabled health technologies among older adults.

## 1. Introduction

Rapidly evolving technological innovations are projected to shape healthcare provision, health management, and the independent living of our growing aging population. Technologies embedded with artificial intelligence (AI) algorithms—such as wearables, robotics, smart home systems, and digital health tools—will transform the ways in which older adults manage chronic conditions and long-term disabilities [1,2]. However, a fundamental unmet concern is whether these technologies are being designed with inclusive and ethical practices that ensure accessibility, usability, privacy, and equity for older end-users, particularly those living with cognitive impairments. Despite their promise, many AI-enabled technologies are developed with minimal involvement from intended end-users, raising concerns about real-world adoption, equity, and ethical implications [2,3]. This article elucidates the concept of co-design within the realm of health technologies and demonstrates how three methodological approaches are being integrated to co-design an innovative smart-home-based socially assistive robot (SAR) for individuals living with Alzheimer’s disease and related dementias (ADRD) and their caregivers.

### 1.1. Background

Market forces continue to drive technological innovations in aging and healthcare. The population of older Americans is projected to reach 80 million by 2040 [4]. The market demand for technologies that support healthy aging and aging in place has never been more significant, owing to the rising prevalence of age-related debilitating conditions; increasing demands on informal caregiving amid a shortage of home health workers; and the surging costs of supervised living facilities. For instance, individuals with ADRD—who experience progressive memory loss, disorientation, and agitation—often require intensive and round-the-clock informal care from family members. As a result, harnessing rapid advances in mainstream AI and consumer electronics to develop a wide range of assistive, rehabilitative, diagnostic, and health-monitoring technologies can support healthy aging and daily functioning while mitigating chronic care burden and stress among informal caregivers.

According to market research, the consumer electronics industry is projected to reach a valuation of $1.78 trillion, with AI and the Internet of Things (IoT) as leading contributors [5]. Within this landscape, the AI in healthcare market is expected to grow exponentially—from $50 billion in 2026 to $700 billion by 2034 [6]. The transformative potential of these mainstream technological innovations to promote independence, health, and social participation among older adults is increasingly recognized [7]. Studies on wearable devices, smart home technologies, telehealth platforms, and digital health tools demonstrate benefits such as real-time health monitoring, early detection, and personalized interventions for chronic disease management [8,9,10,11]. Parallel innovations in assistive and rehabilitation technologies enhance and sustain mobility, cognitive engagement, and personal safety, thereby facilitating aging in place while reducing caregiver burden and healthcare costs [7,12]. Information communication and digital connectivity tools further help combat social isolation by supporting communication, community engagement, and access to services [13,14].

While cost is often considered the primary barrier, limited digital literacy, complex user interfaces, and lack of transparency in automation and decision-making processes also undermine the adoption and usability of these technologies among older adults [15,16,17]. Emerging evidence highlights additional ethical concerns associated with home-based monitoring and AI technologies [3,18]. Our team’s recent scoping review identified key issues including privacy, autonomy, data security, and risks of depersonalization [3]. Consequently, a phenomenon we term the digital paradox emerges: the consumer segment that stands to benefit most from a technology domain is often the least likely to adopt those technologies [19].

End-user involvement in technology development and service design has long been a proven approach to overcoming these barriers. While well-established concepts such as inclusive design and user-centered design help integrate the perspectives of older end-users, these approaches are often characterized as passive, involving minimal or brief interactions with users [20]. In contrast, co-design represents a participatory and iterative approach in which intended end-users and other relevant stakeholders actively contribute throughout the design process—from identifying needs and generating ideas to developing, refining, implementing, and evaluating solutions. The concept originated from participatory design traditions and shares philosophical foundations with participatory action research by emphasizing democratization of the design process, shared decision-making, and recognition of users as experts in their own lived experiences rather than passive recipients of technology [21,22]. Over time, the term has evolved across disciplines and is used alongside related concepts such as participatory design, co-creation, experience-based co-design, and human-centered design, each placing different emphasis on stakeholder roles, stages of involvement, and intended outcomes [23,24,25].

**Figure 1:**
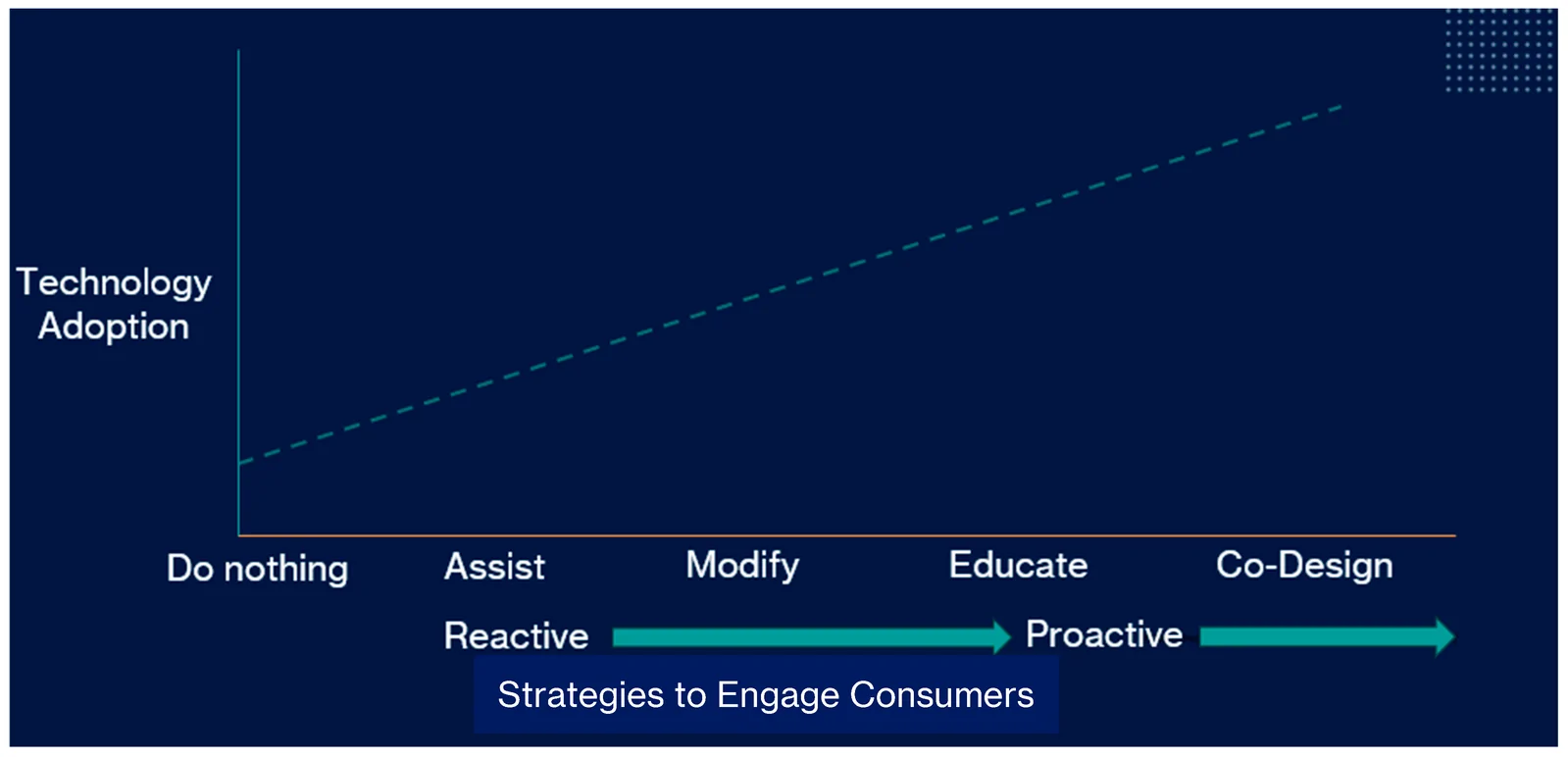
Conceptual Potential of Co-Design on Technology Adoption. The dotted line conceptually predicts the increasing potential of technology adoption as consumer engagement grows with co-design strategy being the most effective.

Consistent with this broader literature, we use co-design to refer to an approach in which older adults and other stakeholders are engaged as active partners across multiple phases of technology development, from ideation through real-world implementation and evaluation. As illustrated in Figure 1, co-design represents a proactive strategy that seeks to identify user needs early, iteratively refine solutions through continuous stakeholder feedback, and evaluate technologies within authentic care environments to maximize ecological validity and long-term adoption. Compared with more reactive approaches—such as modifying fully developed products or relying primarily on user training to compensate for design shortcomings—co-design aims to improve usability, acceptability, and implementation by embedding stakeholder perspectives throughout the development process.

The knowledge base around co-design is evolving, with notable case studies and experts elucidating its application [26,27]. Although co-design has become increasingly prominent in digital health and aging research, there remains no universally accepted definition or standardized methodology. Rather, it is best understood as a family of participatory approaches whose methods vary according to context, stakeholder composition, project goals, and disciplinary traditions [21,24,25]. A systematic review of 34 co-designed technologies and services for older adults similarly found substantial variation in how end-users were involved, the methods employed, and the reported outcomes [20]. Common implementation challenges included establishing meaningful partnerships, balancing stakeholder expertise, managing power dynamics, and ensuring sufficient methodological expertise among research teams.

**Figure 2:**
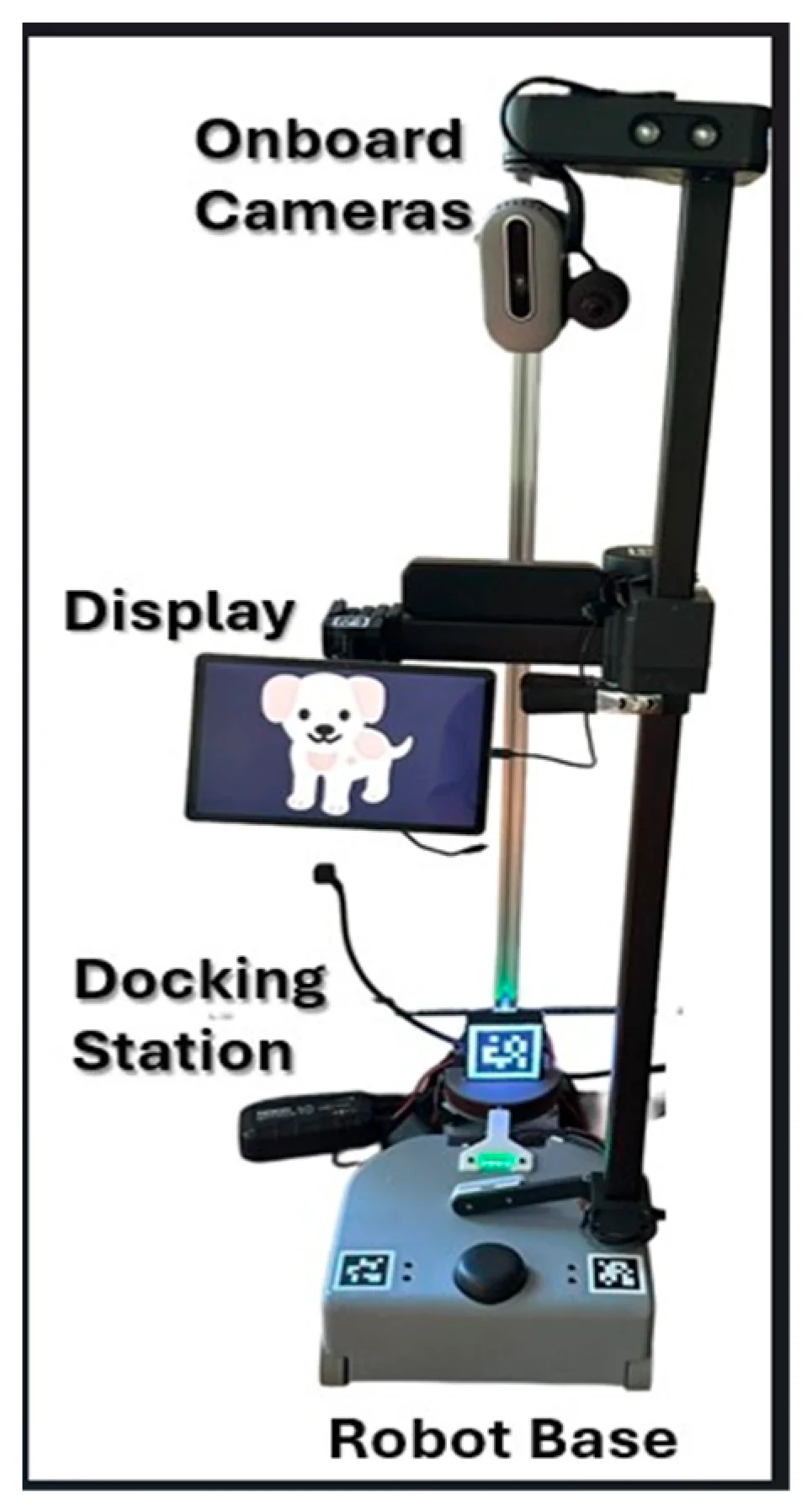
MARSS Prototype.

Therefore, creating, adapting, and demonstrating practical methodologies that exemplify intensive older end-user involvement in emerging AI-based technologies—with implications for health management and independent living—is of paramount importance. This article highlights a three-pronged approach we have integrated to develop and test an AI-enabled SAR for the care of individuals with ADRD in home settings (as shown in Figure 2). Although SARs have been in development for the past two decades, their primary deployment has occurred in supervised living facilities to provide social companionship for older adults [28,29]. Studies also indicate limited development efforts and limited research evidence supporting their long-term deployment for autonomous care of people with ADRD in home and independent living environments [30]. To facilitate successful SAR development, researchers have emphasized the need to understand and contextualize care needs specific to dementia [31]. Therefore, co-designing this technology at the outset is essential.

**Figure 3:**
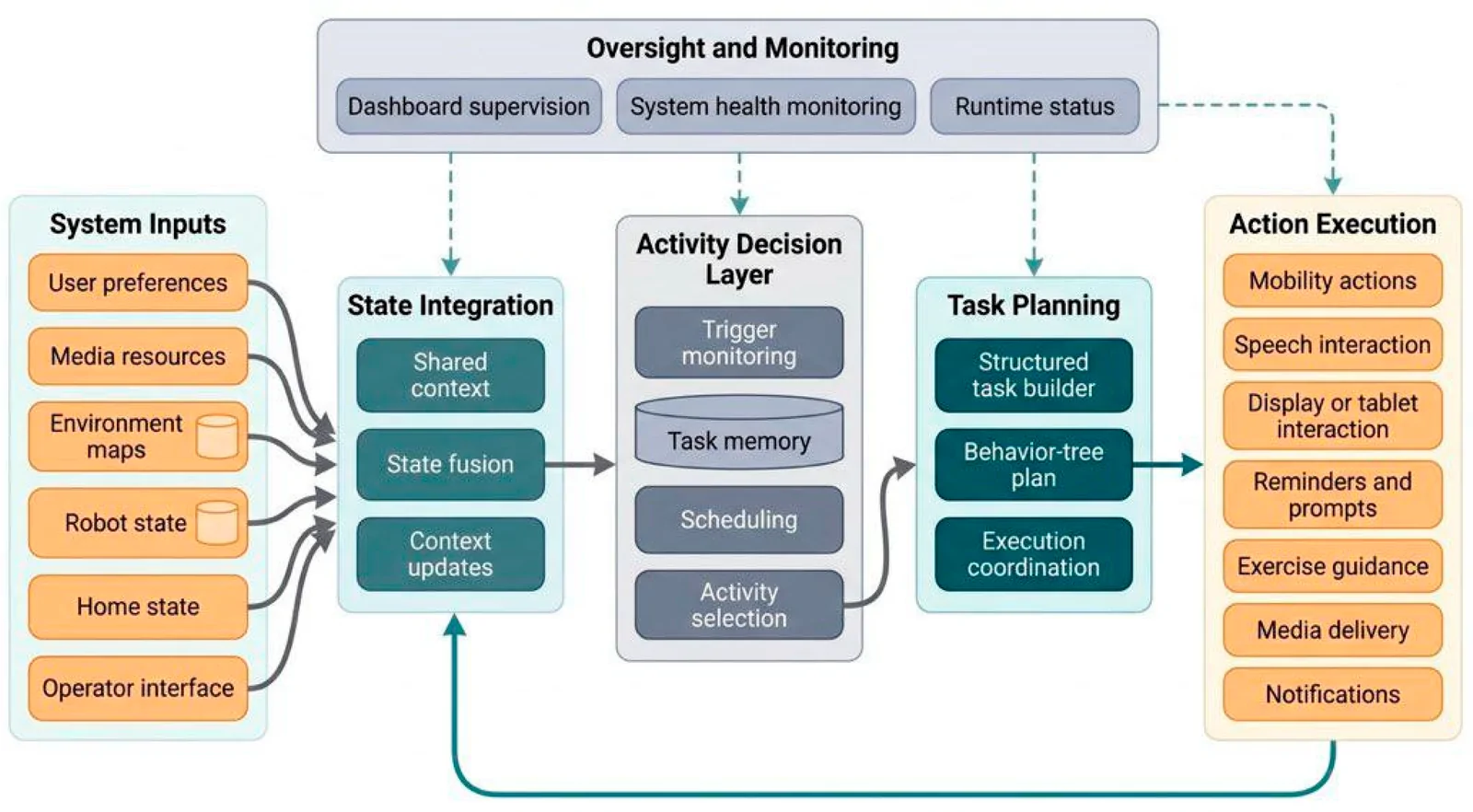
MARSS System Architecture and Functionalities.

Termed the Mobile Assistive Robot with Smart Sensing (MARSS), the framework enables a mobile robot to autonomously deliver a range of care protocols and services while sensing the activities of residents (care recipients and caregivers) and the status of the home environment through distributed sensor networks [32,33]. Figure 3 shows the MARSS system architecture.

The *System Input* to MARSS—consisting of (i) sensor data used to monitor the state of the home, its occupants, and the robot’s current status; (ii) a pre-calculated map of the house and pre-recorded assistive messages (audio and/or video) for the care recipient; and (iii) user-specified guidelines on how MARSS will assist the care recipient—is analyzed in the *State Integration* module to generate the current context of the care task. An example of such context might be: the care recipient is in the living room; the robot is docked; no alarms have been reported by any IoT sensors; and the current time matches the designated time for morning exercise. The *System Input* module leverages state-of-the-art techniques in computer vision and sensor processing.

The *Activity Decision Layer* is a high-level AI planner [34] that continuously analyzes the current context and determines whether a specific care service needs to be triggered. When a care service is required, control is passed to the *Task Planning* module. This module uses behavior trees [35,36]—a type of decision tree—to orchestrate a detailed plan for executing the care service.

Care services—ranging from reminders, alerts, activity assistance (via display monitor), and activity engagement (such as prerecorded exercises)—are delivered fully autonomously through a mobile robot. Our implementation used Stretch 3 mobile robots from Hello Robot Inc., USA, although the MARSS framework is hardware-agnostic. The *Oversight and Monitoring* module performs continuous health checks of all other modules in the MARSS system.

### 1.2. Research Statement

This article demonstrates the application of co-design through an ongoing mixed-methods pilot study to develop and evaluate the feasibility of MARSS with older end-users, individuals with ADRD, and their caregivers. The study integrates the National Institutes of Health (NIH) Stage Model for Behavioral Intervention Development [37] with a proven technology acceptance model, the Unified Theory of Acceptance and Use of Technology (UTAUT) [38], and a client-centric tool, Goal Attainment Scaling (GAS) [39]. UTAUT constructs were used to identify and evaluate determinants of technology adoption, including performance expectancy, effort expectancy, social influence, facilitating conditions, anxiety, and trust. GAS was used to assess individualized outcomes by capturing participant-defined goals and evaluating goal attainment over time.

In conclusion, we propose a technology co-design framework that other developers can use as a model for engaging clinical populations in the development of health technologies.

## 2. Methodology

Our study is part of a pioneering endeavor to develop and deploy SARs to autonomously support the care of individuals with ADRD in their homes and independent living communities. Therefore, co-designing the technology and its integration with end-users is paramount to establishing its acceptance and usability among care partners. Here, we present findings from data gathered from 10 end-users (five caregiver–care recipient dyads) who trialed MARSS in their homes for 1–6 months, with the robot personalized to their condition, home layout, and caregiving needs. To our knowledge, this represents one of the first long-term home deployments of a fully autonomous SAR for ADRD care to date. Our integrated methodological framework is grounded in the NIH Stage Model, with UTAUT and GAS providing real-world perspectives from participants to iteratively refine both the care protocols and the technology.

The NIH Stage Model comprises five stages that we have operationalized as a participatory action approach for technology co-design. Embedded within each stage is the need to examine, engage, and optimize behavioral targets that strengthen the potency of an intervention under development, thereby paving the way for its eventual adoption [37]. Stage 0, *Basic Research*, enables interventionists (in this case, technology developers) to explore mechanisms of action—behavioral maintenance or change—while conceptualizing the technology. Stage I (*Intervention Generation and Refinement*) includes two substages: IA, which focuses on proof-of-concept design with intended behavioral targets in mind, and IB, which involves preliminary efficacy and feasibility testing with end-users or stakeholders [37].

For MARSS, our team previously conducted a Stage IA focus group study demonstrating the first prototype of the SAR to caregivers and care recipients with ADRD and identifying key facilitators and barriers to adoption and acceptance in home environments [32]. In this Stage IB pilot study, our focus is to examine whether real-world exposure and use of MARSS engage those behavioral targets and, importantly, to ensure that technical, logistical, and training refinements are implemented to iteratively strengthen and validate feasibility prior to advancing to the next stage of intervention development.

Stages II and III of the NIH Stage Model are intended for efficacy testing of technologies with high fidelity and internal validity. Stage II is typically implemented by interventionists or technology developers in controlled laboratory settings, while Stage III involves testing the technology in real-world environments by trained personnel or stakeholders [37]. Stage IV emphasizes external validity, assessing the real-world effectiveness of the technology when implemented by clinicians or lay users without expert oversight. Finally, Stage V focuses on dissemination of empirical findings and evidence related to technology adoption and other anticipated outcomes [37].

As is evident, the initial stages of the NIH Stage Model involve low fidelity and offer the most critical co-design window to recursively and iteratively develop the technology in alignment with end-user behavioral targets. The UTAUT and GAS serve as valuable guiding frameworks and tools during this process.

The UTAUT is a well-established framework for technology acceptance, with numerous multivariate studies demonstrating the influence of its constructs on end-user intent to adopt and use technology across multiple domains [40,41,42]. Technology adoption is shaped by the following constructs: (1) *Performance expectancy*, the belief that the technology will help achieve specific goals or fulfill expectations; (2) *Effort expectancy*, the perceived ease or difficulty of using the technology; (3) *Social influence*, the degree to which peers or significant others affect adoption; (4) *Facilitating conditions*, external factors that shape or enable use, including access to devices, reliable internet, and ongoing technical or social support; (5) *Anxiety*, the comfort or discomfort end-users may feel toward the technology; and (6) *Trust*, the perceived dependability or reliability of the technology [38]. Our premise is that each construct elucidates crucial behavioral targets to engage and optimize during the implementation of novel robotics technologies such as MARSS for care recipients and informal caregivers across diverse home settings.

GAS complements our study by verifying preliminary effectiveness of MARSS in terms of technical and clinical feasibility through individualized goal attainment. GAS is a structured scoring method used to create and record participant-specific goals related to an intervention. It is particularly useful for measuring outcomes that are of critical importance to patients or clients in their own contexts when such outcomes cannot be captured through standardized measures [39]. Objective and measurable goals are collaboratively framed along a positive-to-negative continuum. A score of 0 represents expected goal attainment; +1 and +2 indicate outcomes that are *Somewhat better* and *Much better* than expected, respectively; and corresponding negative scores indicate *Somewhat worse* and *Much worse* outcomes. To compute overall goal attainment, each goal is weighted by its importance, the difficulty of achieving it without the technology, and the participant’s baseline score prior to the introduction of the technology [39].

### 2.1. Participants

This study demonstrates a co-design process through data gathered from participant end-users who trialed MARSS for at least one month in their homes. The study protocol was approved by the Institutional Review Board of the University of New Hampshire. The sample includes five older adult dyads (spouses): four caregiver–care recipient dyads in which the care recipients had ADRD, and one dyad without ADRD who volunteered to test our initial prototype during the early phases of the project. Both caregivers and care recipients signed informed consent forms. The consent document included details regarding the setup of the SAR system, including installation of sensors and cameras for monitoring home occupants. Care recipients provided assent when they had difficulty understanding written content.

All dyads consisted of spouses living together—three in a senior retirement facility and two in their own independent homes. Participants with ADRD were in mild to severe stages at the start of their trial, as reported by caregivers based on their most recent neurological evaluations. The key inclusion criterion was that care recipients needed to be able to interact with a SAR, specifically demonstrating the ability to understand and respond to communication or assistance offered, as reported by the caregiver. For individuals in severe stages with minimal interaction potential, care protocols were directed toward caregivers to assist them in day-to-day care tasks. Dyads were excluded if care recipients were non-compliant or had a history of anger or aggression that compromised their safety or the safety of the equipment. Table 1 provides information on the dyads and the care protocols programmed in accordance with their needs.

### 2.2. Data Collection

The primary source of data for this feasibility study was a series of semi-structured interviews conducted immediately following MARSS deployment and at the end of each subsequent month throughout the study. Interviews were guided by a semi-structured interview protocol developed based on the Unified Theory of Acceptance and Use of Technology (UTAUT) constructs, including performance expectancy, effort expectancy, social influence, facilitating conditions, and behavioral intention to use the technology [38]. The guide consisted of core questions asked consistently across all dyads, while allowing flexibility for follow-up probes to explore participants’ unique experiences and emerging issues. Representative questions included: “Can you describe how MARSS has fit into your daily routine?” (performance expectancy); “What aspects of using MARSS worked well or did not work well for you?” (effort expectancy); “What factors made it easier or more difficult to use the robot?” (facilitating conditions); and “How, if at all, has using MARSS influenced your confidence or willingness to continue using assistive technologies in the future?” (behavioral intention).

The interview guide also included questions related to ethical considerations, such as: “Did you ever feel uncomfortable with the robot monitoring activities?”; “Were there situations in which you felt the robot supported or limited your independence or decision-making?”; “Did using MARSS raise any concerns about privacy, trust, or safety?”; and “What recommendations would you make to ensure the robot is used in an ethical and respectful way?”

All interviews were conducted in person by the same trained member of the research team using the standardized interview guide to promote consistency across participants. Interviews lasted approximately 45–90 min depending on the depth of participants’ responses and were audio-recorded and transcribed verbatim. Questions were intentionally phrased using neutral language, and participants were encouraged to discuss both positive and negative experiences with MARSS and explain the reasons underlying their perceptions. Follow-up probing questions were used to clarify responses and obtain richer descriptions while maintaining consistency in the core interview domains. Each dyad also maintained a personal journal to document day-to-day experiences with MARSS. These journals served as memory aids during monthly interviews and were not analyzed as primary data for this study.

Goal Attainment Scaling (GAS) goals were established after MARSS deployment, once dyads had developed a clear understanding of the robot’s setup, intended use, and capabilities. Caregivers formulated individualized goals in consultation with their care recipients based on the care recipient’s abilities and care needs. For each goal, caregivers rated baseline performance (−2 = much below expected to +2 = much better than expected), goal importance (0 = not at all important to 3 = very important), and perceived difficulty (0 = not at all difficult to 3 = very difficult). During each monthly follow-up interview, caregivers reassessed the level of goal attainment using the same five-point GAS scale.

### 2.3. Data Analysis

Qualitative data derived from the UTAUT-guided interviews were analyzed using conventional content analysis [43]. Data collection and analysis occurred concurrently, allowing emerging insights from earlier interviews to inform subsequent discussions while maintaining consistency in the core interview guide. Four members within our research team independently coded the data. They read each transcript multiple times to gain familiarity with the content before conducting open coding. Initial codes were generated inductively from participants’ narratives rather than predetermined, with UTAUT constructs serving as sensitizing concepts to guide interpretation rather than constrain coding.

Following independent coding, the research team met regularly to compare codes, discuss divergent interpretations, and resolve discrepancies through iterative discussion until consensus was reached. Previously coded transcripts were revisited as needed to ensure consistent application of codes across the dataset. Data collection continued until no substantively new concepts or design recommendations emerged, indicating thematic saturation relevant to the objectives of this feasibility study. Codes were subsequently grouped into higher-order categories and synthesized into themes through constant comparison across dyads. Throughout this process, the research team evaluated themes for internal homogeneity—ensuring that data within each theme reflected a coherent underlying concept—and external heterogeneity—ensuring that themes were conceptually distinct from one another.

Goal Attainment Scaling (GAS) outcomes were used descriptively to contextualize participants’ narratives and provide additional insight into factors that appeared to facilitate or hinder progress toward individualized goals. The composite GAS score for each participant was computed using the recommended equation [39]:

$$\mathrm{Overall}\;\mathrm{GAS}\;=\;50+\frac{10\;\Sigma \;(w_{i}X_{i})}{\surd (0.7\;\Sigma \;{w_{i}}^{2}\;+\;0.3\;(\Sigma \;w_{i}{)}^{2})}$$

where w is the weight assigned to the *i*th goal and X the score for the *i*th goal. A post intervention GAS score around 50 is deemed to be a useful quality check in GAS scoring and is considered as a mean T-score relating to accuracy of the goal setting [39].

Rather than serving as independent analytic evidence, GAS findings complemented the qualitative interpretations by illustrating how users’ experiences related to goal attainment and informed subsequent design refinements. In accordance with the aims of this study, the final themes are presented as co-design targets that guided iterative improvements to both MARSS functionality and care protocols.

To enhance analytic rigor and trustworthiness, themes were refined through team consensus and cross-checked against interview transcripts and participant journals to ensure consistency and credibility of interpretation. In addition, preliminary interpretations were discussed with participants during subsequent monthly interviews (member checking), providing opportunities for participants to clarify, confirm, or elaborate on the researchers’ interpretations and ensuring that the findings accurately reflected their lived experiences. The primary purpose of this analysis was to identify user experiences, barriers, facilitators, and co-design targets to inform iterative refinement of the MARSS system. The analysis focused on identifying actionable insights related to usability, acceptability, and real-world integration of the system.

Qualitative findings were used to contextualize and explain patterns in goal attainment, including factors contributing to successful or limited progress. This integration allowed us to link user experiences and barriers to specific design iterations and system refinements implemented during the feasibility study. In accordance with the aims of this study, we present key themes as co-design targets that informed iterative improvements to the technology. Additionally, we examined the underlying reasons for dyads’ attainment or failure to achieve GAS goals as critical inputs for refining both system functionality and care protocols.

## 3. Results

Data analysis resulted in four co-design themes specific to the deployment of MARSS: technical design, care protocol design, training management, and complementary care. Each theme is highlighted in the corresponding table, beginning with the identified behavioral target (category) to engage and optimize, followed by the event or barrier that required resolution or mitigation, and the corresponding design iteration.

Theme 1: Technical Design

As delineated in Table 2, technical problems shaped caregivers’ perspectives on effort expectancy. Early in the deployment, caregivers described navigation issues such as the robot getting stuck on rugs, moving unpredictably, failing to dock, or requiring manual intervention when it lost its path. When the SAR malfunctioned during a task that caregivers had come to rely on, the burden of care immediately resurfaced. In these instances, the robot was no longer reducing workload but instead demanding supervision and troubleshooting. Some caregivers described needing to “baby-sit” the system, which undermined its intended value. These experiences suggest that reliability was central to acceptance: when the SAR functioned smoothly, it was helpful, but when it malfunctioned, it could intensify frustration and reintroduce tasks the caregiver had hoped to delegate.

Related concerns emerged around communication. Prompts could be difficult to hear, especially with background television noise, and some care recipients struggled with the speed or format of the robot’s instructions. Caregivers suggested that a more conversational style and adjustable pacing would better align with the communication needs of people living with dementia.

These insights informed several technical improvements to the system, including substantive enhancements to the robot’s traversability over low-profile obstacles, autonomous docking into its charging station, navigational algorithms, and two-way communication. In addition, we strengthened our capability to remotely monitor the robot’s technical parameters in real time.

Theme 2: Care Protocol Design

Participant narratives provided crucial insights into the need to program care protocols in ways that reflect the nature of ADRD and the daily complexity of caregiving associated with the condition (See Table 3). Rigid, time-based protocols often did not align with the realities of dementia care, where routines can vary considerably from day to day and where individuals with dementia may not respond well to standardized reminders. These dynamics made fixed schedules impractical. In response, we introduced care protocols that were flexible, context-sensitive, and responsive to the person’s status in the moment—for example, initiating morning routines when the care recipient woke up rather than at a predetermined time.

Regardless of whether the technology functioned reliably, caregivers expressed uncertainty about care recipients’ compliance with the robot’s reminders. To address this challenge, we incorporated an activity-recognition algorithm into the daily planner. Certain daily tasks—such as eating, taking medication, or waking up—were distinguished through computer-vision-based kinematics captured from designated cameras. As an additional strategy to ensure completion of high-priority tasks, we created a daily activity checklist that caregivers or care recipients could use to corroborate task completion.

Caregivers were naturally uneasy about the use of cameras and continuous visual monitoring in the home, particularly when monitoring felt excessive or insufficiently justified. These concerns were not rooted in resistance to technology but in issues of dignity and intrusiveness. Caregivers noted that although cameras are increasingly common in public spaces, this does not make them acceptable in intimate domestic environments. Privacy concerns were especially pronounced in private areas such as bedrooms and bathrooms, where caregivers felt that the SAR and its associated sensors could cross important boundaries. Participant narratives made clear that privacy concerns were not minor implementation details; they directly shaped acceptance of the SAR.

Our system was already designed with a local area network for privacy and data storage, selective data processing, and less invasive monitoring approaches—such as sensor-based detection rather than full video capture. However, participant feedback underscored the importance of limiting monitoring to spaces and contexts that are truly necessary, establishing this boundary as a central condition for ethical and practical deployment.

Theme 3: Training Management

As shown in Table 4, our data made clear that not all care partners have the innate readiness to introduce and adopt a SAR in their home. Barriers related to performance expectancy, anxiety, and trust must therefore be explicitly addressed in both training and deployment. One effective strategy we adopted was to introduce care partners to the SAR in our laboratory, demonstrate pertinent use cases and sample protocols, and help them contextualize how the technology might apply to their home environment and naturalistic care scenarios.

The data also reinforced that care protocols, while personalized, are inherently temporal and must adapt to the evolving complexity of ADRD and the associated care burden. The schedule, frequency, pace, and content of reminders, alerts, and activity assistance must remain aligned with the progression of the condition. Caregivers’ comments about trust reflected a similarly measured stance. They did not appear to place unlimited trust in the system, nor did they expect it to enable them to step fully away from care responsibilities. Instead, they viewed the SAR as a tool that could be trusted for selected tasks or limited periods of time, but not as a system capable of independently assuming responsibility for care. This realistic framing is important, as it suggests that caregivers were not rejecting the technology but engaging with it pragmatically and calibrating their expectations to what it could reliably accomplish in practice.

It was also evident that caregivers needed clear and realistic expectations regarding the scope and limits of the SAR. Caregivers consistently expressed that robots could not replace them. They viewed caregiving as fundamentally human and relational, and framed the SAR as a tool that could complement—but not substitute for—the caregiver’s role.

Theme 4: Complementary Care

Findings from the home deployment of the SAR suggest that caregivers generally saw value in the technology when it could meaningfully reduce burden in specific, routine aspects of care (see Table 5). Caregivers described the SAR as most helpful when it supported repetitive tasks such as medication reminders, activity prompts, or other day-to-day caregiving responsibilities that can become exhausting over time. The perceived usefulness of the system was especially strong when it enabled the person living with dementia to continue performing certain activities independently rather than relying entirely on the caregiver. In this sense, the SAR was valued not simply as a reminder tool but as a potential means of preserving function and easing the caregiver’s workload.

Caregivers noted that the system was far less effective when the person with memory loss was resistant, unable to hear the prompts clearly, or had difficulty responding to structured interactions. This highlighted an important condition for successful use: the activity or care protocol had to align with both the interests and the cognitive capabilities of the person receiving care. Caregivers also emphasized that not all tasks were equally important. Medication reminders, for example, were frequently described as a high-priority use case because missed medications could have immediate consequences, whereas other prompts were viewed as more optional or supportive in nature. These distinctions suggest that usefulness was not a general property of the system, but something caregivers evaluated in relation to the importance, feasibility, and reliability of each care task.

### Goal Attainment Results

**Figure 4:**
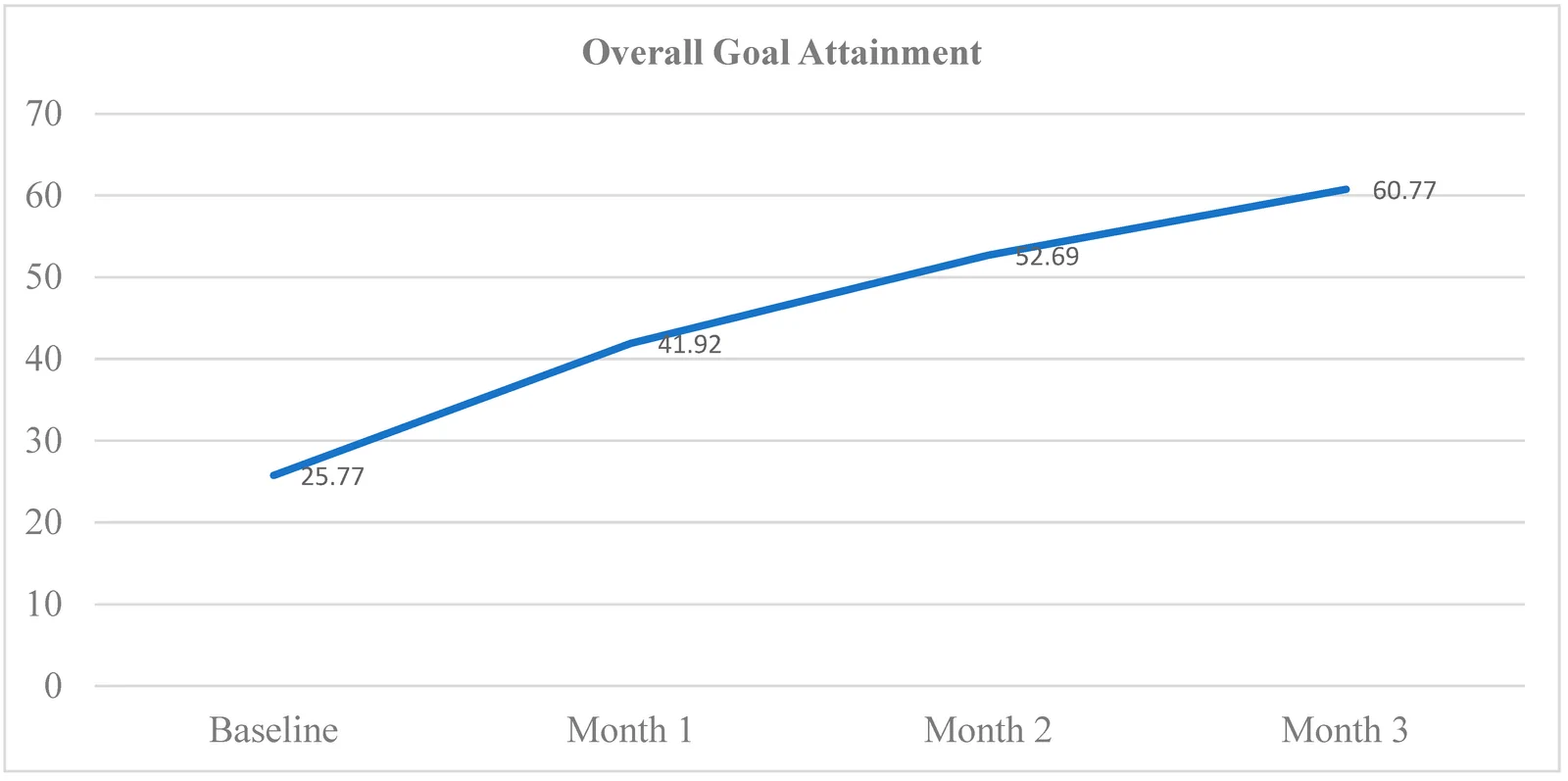
Goal Attainment of Dyad 1.

**Figure 5:**
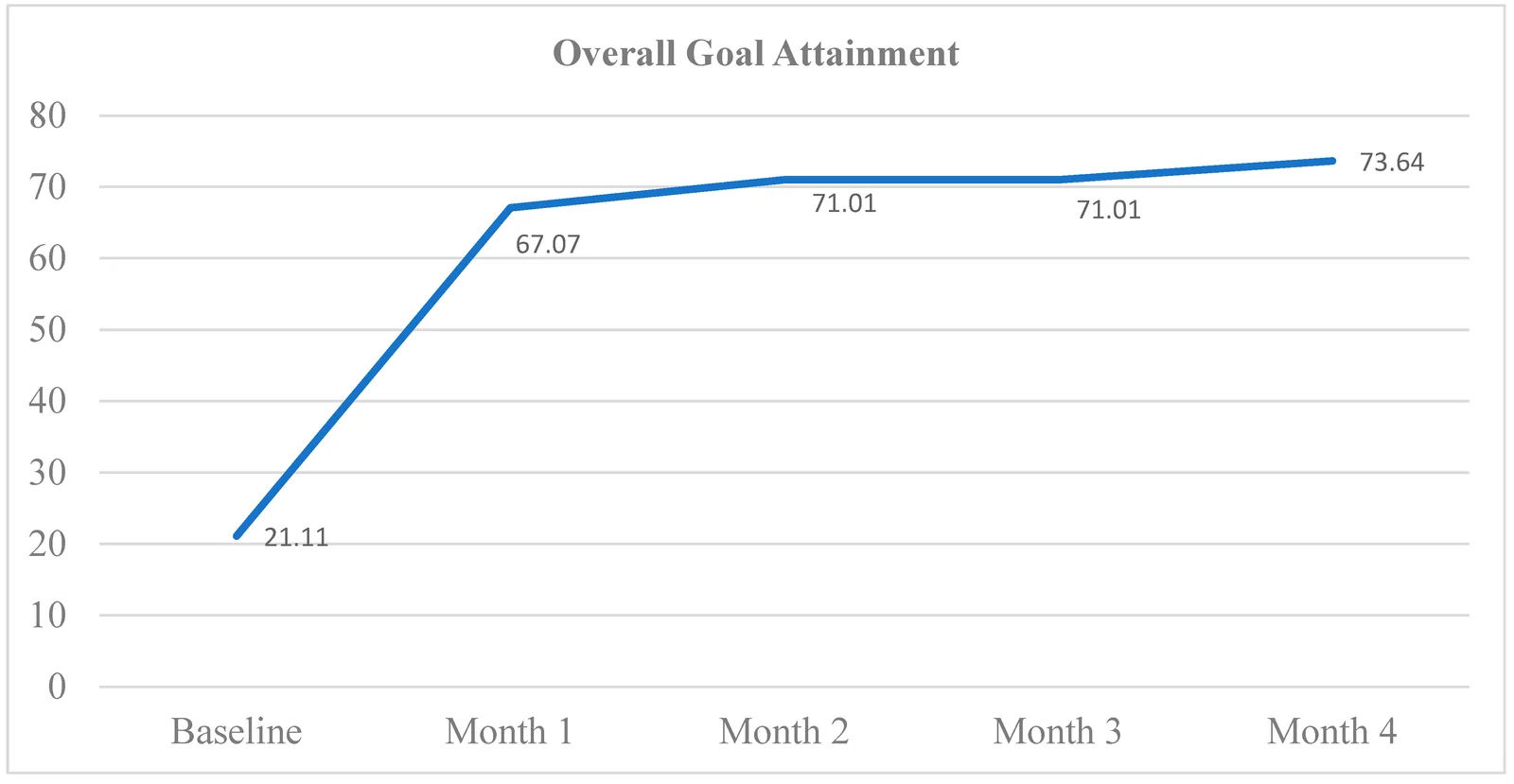
Goal Attainment of Dyad 2.

**Figure 6:**
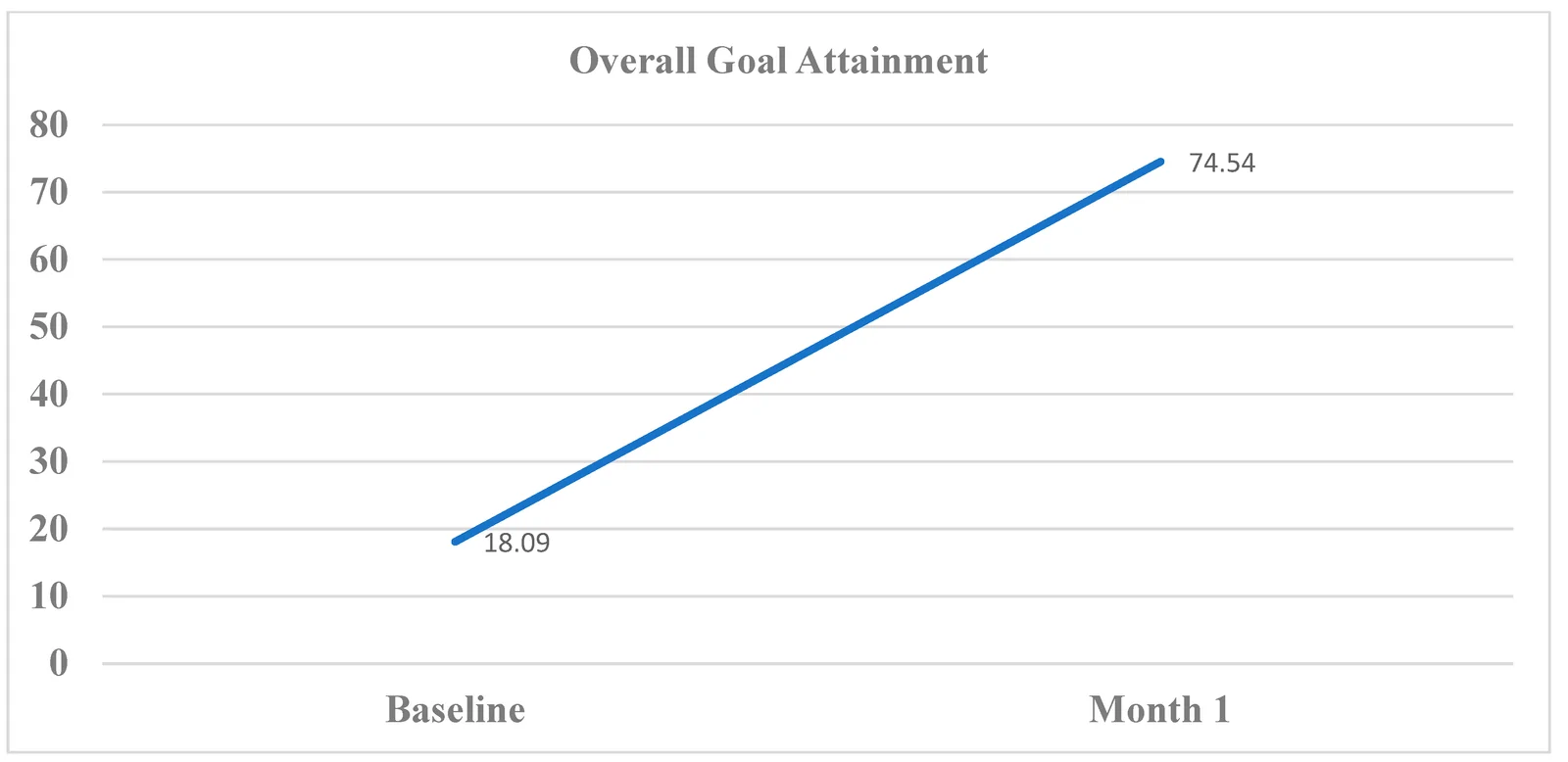
Goal Attainment of Dyad 3.

We present findings from three caregiver–care recipient dyads experiencing ADRD who continued with the trial beyond two months and provided data on their goal attainment. One additional dyad with ADRD discontinued participation due to relocation during the winter months and frequent travel throughout the year, while GAS was not applicable to another dyad that did not have experience with ADRD. Table 6, Table 7 and Table 8 list each dyad’s measurable goals and their corresponding levels of importance, difficulty, and attainment following three months of assistance from MARSS. Figure 4, Figure 5 and Figure 6 illustrate overall goal attainment scores over the course of the trial.

As shown in Figure 4, Dyad 1’s overall goal attainment progressively increased by the end of three months. Consistent with our qualitative findings, goal attainment during the initial months was relatively lower due to technical challenges, care-protocol scheduling issues, unrealistic expectations, and caregiver compliance. Improvements to docking and navigation, adjustments to care protocols (e.g., shifting reminders from fixed times to context-dependent triggers), and modifications to goal expectations (e.g., reducing activity frequency from three to four days per week to once weekly) contributed to the observed gains in goal attainment.

A notable compliance issue early in the trial was that the caregiver often observed the robot delivering protocols but did not consistently act on the reminders. After clarifying that the purpose of the trial was not solely to demonstrate the robot’s technical reliability but also to ensure and promote necessary care support, caregiver compliance improved substantially.

As shown in Figure 5, Dyad II demonstrated substantial goal attainment from baseline through subsequent months. This level of success was attributable not only to three goals being met with performance exceeding expectations, but also to the high importance and difficulty of these goals prior to the SAR’s deployment. In alignment with UTAUT, the dyad’s behavioral intention to rely on the SAR appeared strong from the outset, and the goals themselves were realistic and well-matched to their needs and expectations.

As shown in Figure 6, Dyad 3’s goal attainment was significant within the one-month period during which the SAR was used. Unfortunately, the dyad relocated soon afterward due to the declining health of the care recipient. With respect to medication reminders for the caregiver, optimal goal attainment was achieved when a checklist was displayed on the robot’s touchscreen monitor, allowing her to keep track of multiple medications throughout the day. The care recipient was also at high risk of falling when getting out of bed due to postural hypotension and instability. To address this, a protocol using computer vision linked to the robot was implemented to detect attempts to get out of bed. The robot was positioned near the bedroom to warn the care recipient not to stand and to alert the caregiver when assistance was needed. This protocol underwent rigorous testing in our laboratory prior to deployment and resulted in two true-positive instances in which the care recipient was prevented from getting out of bed.

## 4. Discussion

This study elucidated the co-design process involved in developing an innovative smart-home-based socially assistive robot (SAR) for individuals living with ADRD and their caregivers. As reiterated earlier, the goal was not to evaluate feasibility in a traditional sense, but to highlight three methodological approaches that enabled iterative development of an AI-enabled technology. Ensuring that participants had intensive, long-term, lived experience with the system was essential for an aging-centered technology that must overcome the digital divide. As experts have noted, brief exposures to lab-based prototypes and cursory user input are insufficient for addressing older stakeholders’ limited familiarity, unrealistic expectations, or misinformed skepticism [20,44,45]. Based on the most recent literature on SARs [46,47], this appears to be the first study to co-design a SAR in real-world conditions with individuals living with ADRD and their caregivers. The depth of insight gained was directly attributable to the participatory approach and the lived experience of participants interacting with the technology. The extensive iterations to both the system and its deployment would not have been possible had testing remained confined to controlled settings with minimal user involvement. Continued collaboration with engineers from Hello Robot© further contributed to progressive improvements, underscoring the concerted effort among researchers, technology developers, and end-users in this co-design process.

From a mixed-methods perspective, convergence between qualitative data and GAS outcomes was evident through progressive improvements in goal attainment when care protocols aligned with realistic expectations, when features such as audio communication and system monitoring were added, and when technical reliability improved. Key technical enhancements included safe and reliable navigation across varied home layouts, dependable autonomous docking, improved communication with care partners, strengthened privacy measures, and remote monitoring for timely troubleshooting and maintenance. To enhance interaction, we incorporated a large language model for two-way communication, constrained by a system prompt to function as a concise, helpful robot assistant with guardrails preventing unwarranted conversations or harmful advice. To reinforce privacy, all images, videos, and audio data were processed and discarded locally; only transcribed text of user commands and robot activity was retained, stored locally for debugging and accessible solely to the on-site development team.

These improvements align with findings from prior user-centered studies in which design complexities, interface challenges, and privacy-related apprehensions undermined usability and comfort among older adults interacting with AI-enabled technologies [2,48,49,50,51]. Importantly, the UTAUT model served as a useful complement to the NIH Stage Model, helping validate target behaviors to optimize through design, training, and deployment. Our findings also corroborate prior studies grounded in technology acceptance models, where core constructs such as perceived usefulness, ease of use, anxiety, and facilitating conditions consistently shape adoption among older adults [2,52].

To that end, we learned that care protocols must be realistic, sensitive, and adaptable to the nature of ADRD, and that caregiver presence and involvement remain integral to meaningful use. Comfort and trust must be cultivated through caregiver confidence and self-efficacy, supported by clear demonstrations of the technology’s usefulness. The strongest merits of the SAR were its potential to reduce burden in repetitive tasks, support continued independence, reinforce routines, and serve as a practical adjunct to caregiving. For these benefits to translate into widespread adoption of devices like MARSS, however, issues of effort expectancy identified in this study must continue to be addressed. At the same time, privacy safeguards, safety, technical reliability, emotional comfort, and contextual fit emerged as equally important determinants of acceptability. The findings therefore suggest that successful SAR deployment in dementia care depends not only on what the technology can do, but on whether it can do so reliably, minimally intrusively, ethically, and in ways responsive to the lived realities of caregiving at home. A parallel insight from prior research is that technology integration aligned with caregiver endorsement and established care routines is associated with higher acceptance rates [2,53,54].

### 4.1. Implications

**Figure 7:**
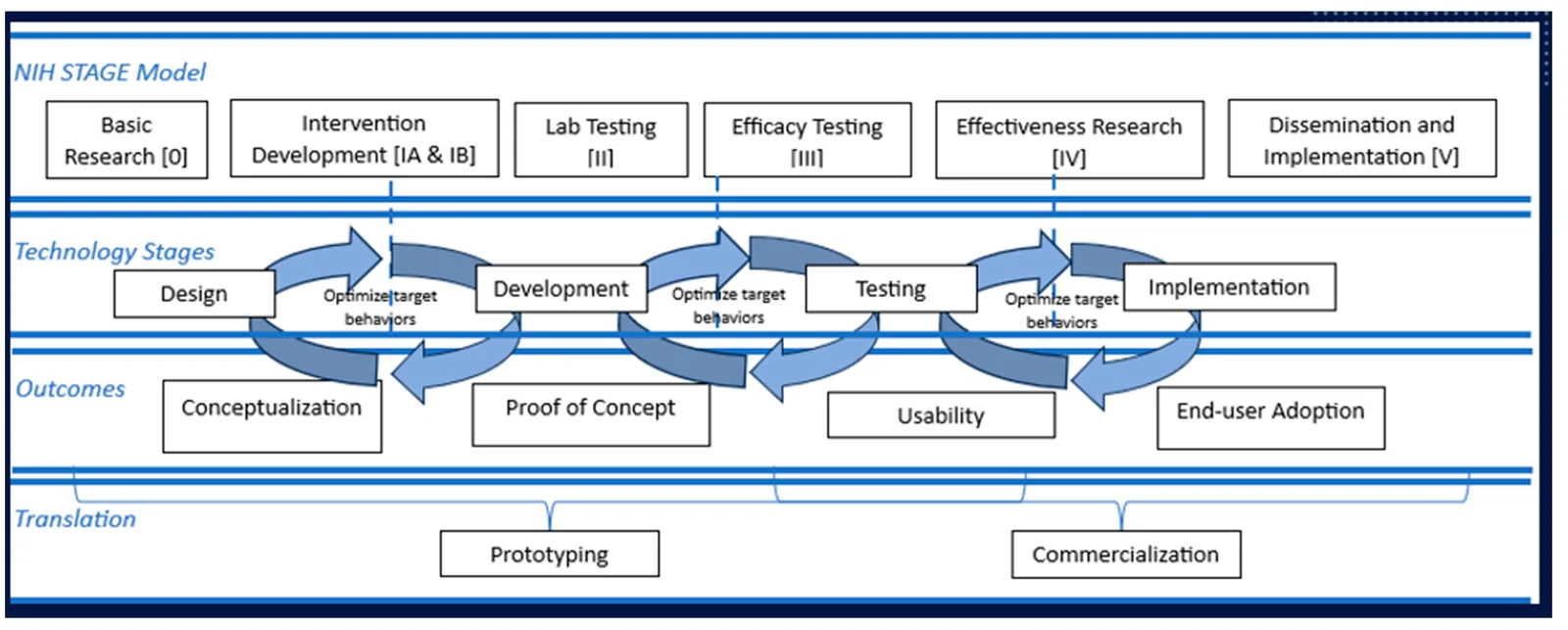
Role of NIH Stage Model for Behavioral Intervention Development in Co-design.

As discussed, multiple methodologies exist for implementing co-design with aging populations, yet outcomes vary widely depending on the nature of the technology, stakeholder inclusion, and level of engagement [20]. A key implication of this research is that the co-design framework we employed is replicable, scalable, and adaptable for future development of digital health innovations, AI-based tools, assistive technologies, and other technology-enabled health interventions. Figure 7 conceptualizes our use of the NIH Stage Model in conjunction with technology-acceptance paradigms to initiate and sustain iterative development of AI-enabled health technologies, illustrating how the co-design process influences technology adoption and translation. The process is iterative and recursive, enabling continuous improvements across design, development, testing, and implementation. Ultimately, the model is intended to augment the potency of the technology, broaden its impact, and enhance clinical fidelity, usability, safety, and end-user adoption [37]. The overarching implication is to strengthen bench-to-bedside translation and commercialization potential, paving the way for consumer satisfaction and improved patient care.

### 4.2. Limitations

This article is based on data derived from an ongoing pilot feasibility study with a small sample of caregiver–care recipient dyads. Although participants engaged intensively and naturalistically with the technology, the duration of their involvement varied considerably—from one to four months—depending on the progression of ADRD. It must also be acknowledged that the care dyads self-enrolled in the study with an inherent interest in robotics and a commitment to contribute to the development of MARSS. As a result, the data are prone to systemic bias, and the experiences and perspectives shared may not be representative of mainstream older consumers. One dyad included in the earliest phase of the pilot study did not have experience with ADRD; however, their engagement and feedback were invaluable for refining the robot’s hardware, mobility, and navigational capabilities, and thus their data were retained in this article.

From a methodological standpoint, although age-centric models such as the Senior Technology Acceptance Model (STAM) have recently been derived from UTAUT [55], the generic UTAUT model was well established and appropriate during the period in which this project was conceptualized.

## 5. Conclusions

The findings of this study indicate that a co-design process that actively engages and optimizes target behaviors can strengthen future adoption of AI-enabled technologies for the aging population. Our study serves as a heuristic for future efforts aiming to co-design technology and enhance adoption. Incorporating theoretically informed, multi-method approaches enables comprehensive data gathering and systematic implementation of co-design recommendations. Iteratively addressing participant feedback during active deployment allows the impact of design changes to be demonstrated within the study period. In particular, combining qualitative insights with objective, observable data such as GAS scores helped substantiate the merits of the co-design process on participants’ goal achievement.

Our ongoing and future work aims to demonstrate the long-term feasibility of MARSS among a broader group of care dyads, alongside evaluating its impact on care relief, daily functioning, and the well-being of care partners.

## Figures and Tables

**Table 1:** Study Participants.

Dyads	Care Recipient Condition	MARSS Care Services/Protocols	Trial Period
Dyad 1 Ages-92 & 88	Moderate AD	Medication Reminders Exercise Reminders Physical Activity Reminders	4 Months
Dyad 2 Ages-65 & 63	Mild AD	Medication Reminders Mealtime Assistance Exercise Reminders Weather Alerts	6 Months
Dyad 3 Ages-78 & 73	Severe Dementia-	Medication Reminders Fall Prevention Alerts	2 Months
Dyad 4 Ages-82 & 80	Moderate AD	Medication Reminders Exercise Reminders Meal Reminders	1 Month
Dyad 5 Ages-78 & 77	No ADRD-Volunteer	Medication Reminders Exercise Reminders Meal Reminders	2 Months

**Table 2:** Technical Design.

Behavioral Target	Barrier/Data Insight	Illustrative Quote [Dyad#]	UTAUT Construct	Iterations
Safe navigation	Robot gets stuck on rugs and obstacles	“It would get stuck on the rugs and…run around in circles.”[D5]	Effort Expectancy	Improved navigation and mechanical capability for lower profile obstacles
Reliable docking	Docking failures require caregiver interventions	“I worry when he doesn’t dock himself.”[D2]	Effort Expectancy	Added infrared sensing alongside April tag for docking
Predictable movement	Unpredictable motion raises safety concerns	“The motion can be unpredictable at times.”[D5]	Effort Expectancy	Constrained robot to consistent, predictable paths
Collision avoidance	Robot collides with objects at home	“It has run into things…had to pause it.”[D5]	Effort Expectancy	Repeated testing refinement of navigation algorithm
Audio communication	Users cannot hear robot clearly	“Volume would drop and hard to hear”[D1]	Effort Expectancy	Integrated external microphones and speakers
System monitoring	Failures not detected in real time	“There should be a signal…to respond immediately.”[D1]	Effort Expectancy	Added remote data logger for system monitoring
Aesthetics	Anthropomorphism is not a great priority	It doesn’t matter what it looks like if it does it’s job” [D3]	Performance Expectancy	Functionality and usability take precedence in the design
Privacy acceptance	Camera use raises concerns	“There’s some loss of privacy.”[D1]	Anxiety	Local data storage and selective data processing
Technological disruptions	System failures during outages	“Power failure caused it to pause.”[D1]	Facilitating Conditions	Developed remote power monitoring and troubleshooting guide

**Table 3:** Care Protocol Design.

Behavioral Target	Barrier/Data Insight	Illustrative Quote [Dyad#]	UTAUT Construct	Iterations
Unpredictability of daily routines	Time-based reminders are ineffective	“I don’t trouble her if she’s sleeping.”[D1]	Effort Expectancy	Shifted to context triggered or person-triggered protocols
Protocol compliance	Difficulty confirming task completion	“How is it…determined a medication was taken?”[D1]	Effort Expectancy	Integrated an activity recognition algorithm
Task prioritization	Not all care tasks are equally important	“Medication is probably the most obvious one. Everything else is sort of voluntary.’[D1]	Performance Expectancy	Added compliance checks for high-priority tasks
Monitoring in private spaces	Cameras not acceptable in bedrooms/bathrooms	“We decided...it was not appropriate.”[D4]	Anxiety	Replaced cameras with motion and door sensors
System placement safety	Robot may create fall risks	“I don’t want to trip over the robot at night.”[D5]	Anxiety	Added visual indicator and safer docking placement
Home layout variability	Changes in environment disrupt navigation	“Housekeeping moved our furniture.” [D5]	Facilitating Conditions	Clarified navigation paths and set up requirements
Communication style	Structured commands reduce compliance	“Having two-way communication might be helpful.”[D1]	Effort Expectancy	Shifted to conversational interactions

**Table 4:** Training Management.

Behavioral Target	Barrier/Data Insight	Illustrative Quote [Dyad#]	UTAUT Construct	Iterations
Technology acceptance	Users may feel intimidated by robot	“Some people [may] be intimidated.”[D1]	Anxiety	Introduced pre-deployment demos and interactions
Clarification of caregiver role	Concern that robot replaces caregiving	“Caregiving is done by people.”[D3]	Performance Expectancy	Framed SAR as complementary to caregiving roles
Future readiness	Caregivers anticipate declining ability	“It could be valuable…if I started to have memory problems.”[D1]	Performance Expectancy	Designed protocols to support caregiver needs over time
Safety monitoring	Realistic degree of trust	“I don’t expect to leave him with the robot for three days”[D2]	Trust	Establish what the robot could be relied upon
Assistance pacing	Information delivered too quickly for user processing	“He struggled with the speediness of the video.”[D2]	Effort Expectancy	Adjusted pacing of instructions based on cognitive needs

**Table 5:** Complementary Care.

Behavioral Target	Barrier/Data Insight	Illustrative Quote [Dyad#]	UTAUT Construct	Improvement Implemented
Care recipient engagement	Resistance to robot-directed tasks	“You can’t do it if the person…is resistant.”[D4]	Performance Expectancy	Matched protocols to user interests and abilities.
Care respite	Caregivers overwhelmed by routine tasks	“It’s those repetitive trivial tasks that drive us crazy.” “And if only one less thing to do”[D2]	Performance Expectancy	Automated, simple repetitive care tasks
Routine formation	Habit formation varies across users	“I’m getting more habitual in responding to the robot.” [D2]	Effort Expectancy	Care protocols to be maintained, simplified, weaned off or graded up
Role & identity	Unrealistic expectations of autonomy	“I don’t think the robot is yet able to replace me.”[D3]	Performance Expectancy	Reinforced assistive role of SAR, not replacement

**Table 6:** Goals for Dyad 1.

Care Service	Goal to Achieve After the Care Service	Importance	Baseline Difficulty	Goal Attainment [at 3 Months]
Medication reminder	Caregiver administers meds	3-Very Important	1-Little Difficulty	+1 Administers all meds throughout the week
Exercise reminder	Caregiver exercises with the care recipient	2-Moderately Important	3-Very Difficult	0 Performs exercises together once or twice a week
Walking reminder during warm weather	Caregiver takes care recipient on a walk	2-Moderately Important	3-Very Difficult	+1 Goes for walk once or twice a week

**Table 7:** Goals for Dyad 2.

Care Service	Goal to Achieve After the Care Service	Importance	Baseline Difficulty	Goal Attainment [at 4 Months]
Assistance using appliances	Care recipient will independently use coffee maker or microwave watching a video on robot’s display	3-Very Important	2-Somewhat Difficult	0 Can make coffee 3–4 days a week without assistance
Medication reminder	Care recipient takes medications on time without assistance	3-Very Important	3-Very Difficult	+1 Takes meds without caregiver’s prompts most of the time
Reminders before walking	Care recipient ensures that proper attire is worn before walking	3-Very Important	3-Very Difficult	+2 Wears proper attire all the time without the caregiver prompting
Trash pick-up	Care recipient will pick and leave trash outside once a week	3-Very Important	3-Very Difficult	+2 Does it all the time following the reminder

**Table 8:** Goals for Dyad 3.

Care Service	Goal to Achieve After the Care Service	Importance	Baseline Difficulty	Goal Attainment [at 4 Months]
Medication reminder	Caregiver administers meds	3-Very Important	2-Somewhat Difficult	+2 Administers all meds throughout the week
Wake up alert (PT)	Care recipient is alerted from not getting up and caregiver is notified	3-Very Important	3-Very Difficult	+2 Care recipient prevented from getting up each time

## Data Availability

Data collected in this study is unavailable due to confidentiality agreement, privacy and ethical restriction.
